# Results of advanced core decompression in patients with osteonecrosis of the femoral head depending on age and sex—a prospective cohort study

**DOI:** 10.1186/s13018-020-01643-4

**Published:** 2020-03-31

**Authors:** Sebastian Serong, Marcel Haversath, Tjark Tassemeier, Florian Dittrich, Stefan Landgraeber

**Affiliations:** 1 Department of Orthopaedics & Orthopaedic Surgery, Saarland University, Kirrberger Strasse 100, 66421 Homburg, Germany; 2 Department of Orthopaedics & Traumatology, University of Duisburg-Essen, Essen, Germany

**Keywords:** Osteonecrosis, Hip, Core decompression, Age, Sex

## Abstract

**Background:**

Core decompression is a common surgical technique to treat osteonecrosis of the femoral head. The aim of this study is to evaluate the effect of the parameters “age” and “sex” on the outcome of this type of treatment.

**Methods:**

A prospective cohort study was performed. Eighty-six osteonecrotic hips with a mean follow-up of 32.5 months (± 24.8) after advanced core decompression were analysed regarding age- and sex-dependent treatment failure. Additionally, the modified Harris Hip Score and Numeric Rating Scale were compared regarding the parameters age and sex.

**Results:**

The mean hip survival of the male participants was 51.3 months (39.4% treatment failure), whereas females presented a longer, thus not significant, mean survival of 61.4 months (30% therapy failure; *p* = 0.48). The further evaluation revealed significantly better survival in the patients aged < 40 years (mean survival 66.09 months, 16% treatment failure) in comparison to those aged ≥ 40 years (mean survival 50.14 months, 46% therapy failure; *p* = 0.03). The modified Harris Hip Score and Numeric Rating Scale results of patients whose treatment did not fail during the study period were similar, irrespective of the patient’s sex or age.

**Conclusions:**

The study shows that the number of therapy failures is significantly higher in older patients, with 40 years of age marking the borderline. Patients’ sex does not seem to affect the outcome of treatment, and postoperative clinical scores appear to be identical with individuals not affected by therapy failure. Since age and sex are unalterable parameters, the study helps to provide valuable predictions regarding the chances of long-term hip survival after treatment of osteonecrosis.

## Background

Osteonecrosis of the femoral head (ONFH) is a pathological condition of the hip joint characterized by impaired blood supply which induces progressing structural instability. Missed or delayed treatment regularly results in collapse of the femoral head with arthroplasty being the only remaining treatment option [1, 2]. It is supposed that ONFH is based on a multifactorial genesis of which several risk factors such as corticosteroid treatment, coagulopathies and heavy alcohol and nicotine consumption have been identified [3, 4]. The average prevalence is estimated to be 29 out of 100,000 each year with a peak in age distribution between 30 and 50 years. A further typical finding is the predominant occurrence of ONFH in the male sex [5, 6].

As ONFH harbours the severe risk of irreversible damage to the affected hip, it is essential that treatment should be commenced as soon as possible. Non-surgical options have been described for treatment of early-stage ONFH that is often characterized by diffuse bone marrow oedema [7–9]. In that context iloprost, enoxaparin and bisphosphonate treatment have been reported [10–12]. Moreover, recent studies showed extracorporeal shock wave therapy to be a further promising approach in the management of early-stage ONFH [13–15].

However, to the present day, surgical procedures still are the therapy of choice in most cases, especially when necrotic zones have begun to demarcate [3, 16]. Several techniques have been published with core decompression and its variations being one of the most frequently performed procedures. All types of core decompression aim at pain reduction and long-term preservation of the femoral head provided that it has not yet collapsed [3, 17]. Although differences in the rate of hip survival have been reported depending on the type of procedure performed, there are further factors that distinctly limit therapy success. Treatment of advanced ONFH stages as described in the ARCO and Steinberg classifications is more likely to fail than treatment of early ones [3, 18–20]. Lesion size also seems to have a significant effect on outcome, with larger lesions often resulting in joint-line collapse despite surgery [21]. Concomitant pathologies of the hip joint such as the femoroacetabular impingement syndrome seem to reduce the rate of therapy success in ONFH even further [22]. Furthermore, therapy is also more likely to be jeopardised if known risk factors such as ongoing corticosteroid treatment or addictive alcohol consumption cannot be eliminated.

As for the parameters “age” and “sex”, their influence on ONFH therapy outcome has so far not been sufficiently evaluated. Shimizu et al. were able to show that alcohol-induced ONFH was more often triggered in male rats than in females and thereby supposed the presence of unknown sex-based factors for its predominant occurrence in men [23]. However, they did not provide a prognosis concerning therapy outcome depending on sex. The same applies for the parameter “age”. We know the age distribution for the occurrence of ONFH but nothing about its influence on therapy success. This study therefore aims to present the age- and sex-dependent outcome of ONFH therapy through advanced core decompression.

## Methods

A prospective, non-randomized design was chosen (level II-prospective cohort study). The study was approved by the institutional review board of the University of Duisburg-Essen, and informed consent was obtained from all participants. Conventional x-ray and magnetic resonance imaging were used to diagnose ONFH. In order to avoid a stage-dependent outcome bias, only patients with ARCO and Steinberg stage 2 lesions were included [20, 24]. All patients underwent detailed preoperative physical examination and were followed up regularly at the Department of Orthopaedics and Traumatology of the University Hospital Essen. The recorded parameters included the modified Harris Hip Score (mHHS) for evaluation of hip function and the numeric rating scale (NRS) for pain quantification [25, 26]. ONFH was treated solely by advanced core decompression (ACD) and its modified version (mACD) between the years 2011 and 2016 as described by Landgraeber et al. [17, 27]. Both methods are characterized by fluoroscopic-guided drilling into the defect zone and removal of the necrotic tissue using an expandable reamer. Whereas intraosseous defects were filled solely with synthetic bone substitute composed of calcium sulfate (CaSO_4_)/calcium phosphate (CaPO_4_) in conventional ACD, a mixture of autologous cancellous bone from the femoral neck and the same synthetic bone substitute was used in cases of mACD.

The study collective included 71 patients with ONFH resulting in a total number of 86 affected hips due to 15 cases of bilateral occurrence. The follow-up period was termed for 2 years with postoperative assessment points set at 6 weeks, 6 months, 12 months and 24 months. As numerous patients continued to regularly present themselves, a longer overall follow-up period of 32.5 months was calculated. The primary objective of the study was to evaluate the long-term results of ONFH treatment by ACD considering the parameters “age” and “sex” in particular. In order to assess the treatment outcome, subgroups on the basis of these two specific parameters were formed. Detailed baseline characteristics of the entire study collective as well as the created subgroups are presented in Table 1 and Table 2.

**Table 1:** Study collective’s baseline characteristics relating to the total number of hips affected by ONFH

Study collective—ONFH-hips total	*N* = 86
ONFH-occurrence (count), monolateral/bilateral	56/15
Sex (count), male/female	66/20
Age (years), mean ± SD/min./max.	46.8 ± 12.2/21.8/69.7
Treatment type (count), ACD/mACD	46/40
Follow-up (months), mean ± SD	32.5 ± 24.8

*SD* standard deviation

**Table 2:** Baseline characteristics of the formed age- and sex-dependent ONFH-subgroups

Subgroup “Age”	ONFH, mono-/bilateral (count)	Sex, male/female (count)	Age, years (mean ± SD)	Treatment, ACD/mACD (count)	Follow-up, months (mean ± SD)
**< 30 years** (*N* = 9)	8/1	6/3	n.a.	5/4	33.41 ± 24.61
**≥ 30/< 40 years** (*N* = 16)	11/5	10/6	n.a.	7/9	39.18 ± 26.26
≥ **40/< 50 years** (*N* = 27)	23/4	25/2	n.a.	15/12	34.49 ± 24.11
**≥ 50 years** (*N* = 34)	29/5	25/9	n.a.	19/15	27.44 ± 24.90
**Subgroup “Sex”**					
**Male** (*N* = 66)	52/14	n.a.	47.34 ± 11.74	33/33	29.67 ± 23.15
**Female** (*N* = 20)	19/1	n.a.	45.16 ± 13.65	13/7	41.68 ± 28.49

*SD* standard deviation

We proposed the hypothesis that the higher a patient’s age, the lower the chances of therapy success, whereas the patient’s sex does not affect therapy results. The primary study endpoint was defined as the need for arthroplasty due to radiological evidence of collapse of the femoral head. The secondary endpoint was set as the evidence of age- or sex-dependent differences in joint function or pain level by analysing the postoperative mHHS and NRS scores.

As it is known that the treatment outcome of ONFH depends on several co-factors, the subgroups were further analysed regarding the presence and distribution of possible confounders. The parameters recorded in this context include the size of the necrotic lesion (A–C according the ARCO and Steinberg classifications), the presence of risk factors (immunosuppressive therapy, high-dose corticosteroid treatment, abuse of alcohol and nicotine and coagulation disorders) and radiological evidence of cam-type deformity of the affected hip using the alpha angle according to Nötzli [21, 22, 28, 29].

Statistical analysis was performed using SPSS® Statistics (Version 21.0, IBM®). Concerning the primary study endpoint, a Kaplan-Meier survival analysis was performed separately for the parameters “age” and “sex”. The log-rank test was used to examine significant statistical differences. Significance level was set at *p* < 0.05 (significant). For comparison of pre- and postoperative mHHS and NRS (secondary study endpoint) sample distribution was tested using the Kolmogorov-Smirnov test. In case of normal distribution, the *t* test for independent and dependent parametric samples was used; otherwise, the Wilcoxon rank-sum test was utilized. Patients with treatment failure in the postoperative course were not considered. Regarding the influence of possible confounders, cross tables combined with the chi-square test were used to describe their distribution in the subgroups and thereby check for relevant differences.

## Results

### Primary study endpoint—treatment failure

**Fig. 1:**
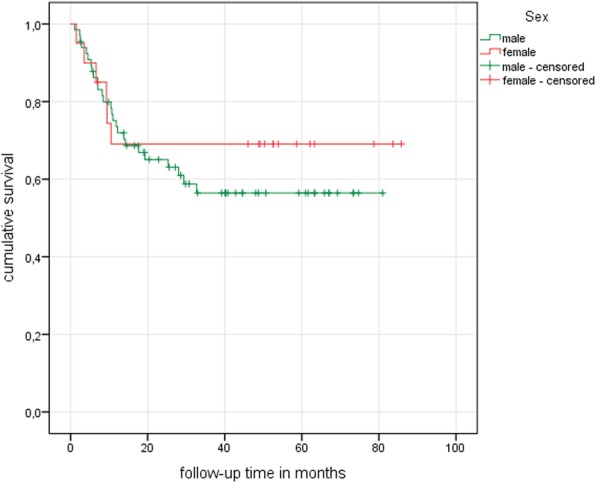
Kaplan-Meier curve showing sex-dependent hip survival

In the study collective as a whole, collapse of the femoral head and thereby failure of treatment was identified in 32 hips (37.2%) during the follow-up period. As regards the patients’ sex, treatment failed in 26 out of 66 male hips (39.4%) after an average of 11.15 (± 8.87) months. For the female participants, treatment failure was registered in 6 out of 20 hips (30%) after an average 6.33 (± 3.68) months **(**Table 3**)**. Using the Kaplan-Meier survival estimator, a mean survival time of 51.3 months was calculated for the male hips, while the mean survival of the female hips was 61.4 months. However, despite the better survival of the female hips, the differences were not statistically significant in the log-rank test (*p* = 0.48) **(**Fig. 1**)**.

**Table 3:** Age- and sex-dependent therapy outcome in total numbers and rounded percentages

Study collective		Therapy failure (count and percentage)		
		No	Yes	Subgroup total
**Subgroup 1** (< 30 years)	**Total** male/female	**7 (77.78%)** 5/2 (55.56%/22.22%)	**2 (22.22%)** 1/1 (11.11%/11.11%)	**9 (100%)** 6/3 (66.67%/33.33%)
**Subgroup 2** (≥ 30/< 40 years)	**Total** male/female	**14 (87.50%)** 9/5 (56.25%/31.25%)	**2 (12.50%)** 1/1 (6.25%/6.25%)	**16 (100%)** 10/6 (62.50%/37.50%)
**Subgroup 3** (≥ 40/< 50 years)	**Total** male/female	**16 (59.26%)** 14/2 (51.85%/7.41%)	**11 (40.74%)** 11/0 (40.75%/0%)	**27 (100%)** 25/2 (92.59%/7.41%)
**Subgroup 4** (≥ 50 years)	**Total** male/female	**17 (50.00%)** 12/5 (35.29%/14.71%)	**17 (50.00%)** 13/4 (38.24%/11.76%)	**34 (100%)** 25/9 (73.53%/26.47%)
Total		54 (62.79%)	32 (37.21%)	86 (100%)

**Fig. 2:**
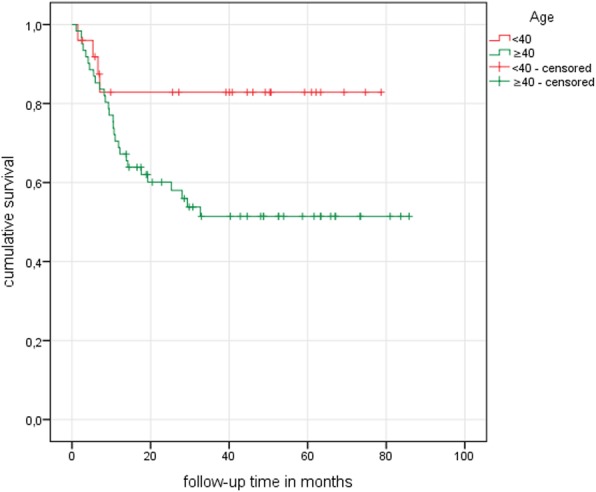
Kaplan-Meier curve showing age-dependent hip survival

Regarding the parameter “age”, therapy failure was documented in 2 cases (22.22%) in subgroup 1 (< 30 years) after an average of 4.00 (± 4.24) months. Subgroup 2 (≥ 30/< 40 years) also had 2 cases (12.50%, 5.50 months ± 0.71) of treatment failure, whereas subgroups 3 (≥ 40/< 50 years) and 4 (≥ 50 years) presented with 11 (40.47%, 14.91 months ± 11.47) and 17 (50%, 8.53 months ±5.00) unsatisfactory therapy results respectively **(**Table 3**)**. As the differences in hip survival between subgroups 1 and 2, as well as between subgroups 3 and 4 were not significant during the observation period; a cut-off was made at the age of 40. By means of Kaplan-Meier survival analysis, a mean hip survival of 66.09 months was calculated for study participants younger than 40 years of age. Study subjects older than 40 showed a calculated mean survival of 50.14 months. The log-rank test showed these differences to be significant with *p* = 0.03 (Fig. 2).

### Secondary study endpoint—clinical outcome

Evaluation of the preoperative mHHS and NRS data revealed an overall preoperative mean mHHS of 69.93 (± 13.81) and a mean NRS of 3.79 (± 2.14). Overall postoperative results showed a significant improvement with the mean final mHHS at 86.13 (± 7.99) and mean final NRS at 0.96 (± 1.52) at the final postoperative assessment (*p* < 0.001 each).

Concerning the sex-based subgroups, the mHHS of the male participants improved from 71.39 (± 13.92) to 86.70 (± 7.82) and the NRS from 3.59 (± 2.22) to 0.75 (± 1.13) over the study period. For the female study patients, an improvement in the mHHS from 65.10 (± 12.60) to 84.50 (± 8.55), and in the NRS from 4.45 (± 1.73) to 1.57 (± 2.24) was seen. Both subgroups showed a similar increase in the mean mHHS (15.31 vs. 19.40) and a similar decrease in the mean NRS (2.84 vs. 2.88) during follow-up with inter-subgroup differences not being significant (*p* > 0.05). Similar changes were also to be seen at the corresponding follow-up times (Table 4). It must be mentioned that despite a similar overall improvement, the female participants had a distinctly higher pre- and postoperative NRS score in comparison to their male counterparts.

**Table 4:** Pre- and (final) postoperative age- and sex-dependent changes in mHHS and NRS

Subgroup “Age”	mHHS (mean ± SD)		NRS (mean ± SD)	
	preoperative	postoperative	preoperative	postoperative
**Subgroup 1** (< 30 years)	69.67 ± 13.39	84.86 ± 8.01 FU1 88.25 ± 1.89 FU2 89.25 ± 1.26 FU3 87.40 ± 1.67 FU4 83.50 ± 8.57	3.56 ± 2.13	1.00 ± 1.16 FU1 0.75 ± 0.96 FU2 0.50 ± 0.58 FU3 0.80 ± 0.84 FU4 1.00 ± 1.10
**Subgroup 2** (≥30/<40 years)	72.31 ± 12.65	87.07 ± 5.21 FU1 83.92 ± 8.14 FU2 87.67 ± 3.72 FU3 86.50 ± 1.00 FU4 86.00 ± 5.07	3.75 ± 2.30	0.93 ± 1.33 FU1 1.00 ± 1.16 FU2 0.83 ± 0.98 FU3 0.75 ± 0.96 FU4 0.86 ± 1.22
**Subgroup 3** (≥ 40/< 50 years)	72.19 ± 14.61	86.76 ± 8.93 FU1 85.36 ± 8.13 FU2 88.14 ± 4.45 FU3 89.00 ± 4.87 FU4 87.08 ± 8.14	3.37 ± 2.36	1.12 ± 2.12 FU1 0.91 ± 1.14 FU2 0.43 ± 0.54 FU3 0.50 ± 0.76 FU4 1.57 ± 2.24
**Subgroup 4** (≥ 50 years)	67.09 ± 13.84	85.19 ± 9.40 FU1 88.00 ± 4.99 FU2 84.00 ± 8.76 FU3 87.56 ± 4.50 FU4 87.10 ± 5.13	4.21 ± 1.89	0.81 ± 1.11 FU1 0.43 ± 0.65 FU2 0.90 ± 0.99 FU3 0.56 ± 0.73 FU4 0.70 ± 1.06
**Subgroup “Sex”**				
**Male**	71.39 ± 13.92	86.70 ± 7.82 FU1 86.45 ± 6.39 FU2 86.37 ± 6.74 FU3 87.71 ± 4.08 FU4 86.22 ± 6.35	3.59 ± 2.22	0.75 ± 1.13 FU1 0.83 ± 1.10 FU2 0.74 ± 0.81 FU3 0.57 ± 0.75 FU4 1.07 ± 1.51
**Female**	65.10 ± 12.60	84.50 ± 8.55 FU1 85.23 ± 8.01 FU2 87.38 ± 5.07 FU3 88.20 ± 2.68 FU4 86.38 ± 8.47	4.45 ± 1.73	1.57 ± 2.24 FU1 0.62 ± 0.65 FU2 0.63 ± 0.92 FU3 0.80 ± 0.84 FU4 1.11 ± 1.63
**Total**	**69.93 ± 13.81**	**86.13 ± 7.99**	**3.79 ± 2.14**	**0.96 ± 1.52**

*SD* standard deviation, *FU1* follow-up 6 weeks, *FU2* follow-up 6 months, *FU3* follow-up 12 months, *FU4* follow-up 24 months

**Fig. 3:**
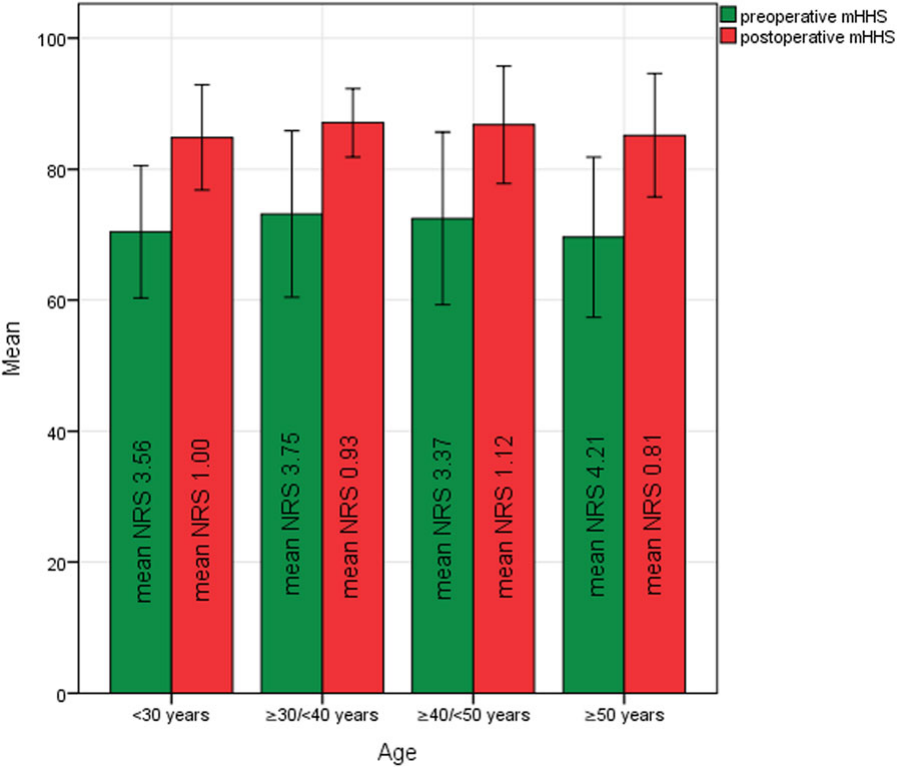
Bar graph illustrating similar age-dependent changes in pre- and postoperative mHHS and NRS for non-collapsed hips (error bar 1 STD)

As for the parameter “sex”, the alterations in mHHS and NRS were analogical in the determined age-dependent subgroups. All subgroups (1 (<30 years), 2 (≥30/<40 years), 3 (≥40/<50 years) and 4 (≥50 years)) showed a similar increase in mHHS, accompanied by a corresponding decrease in NRS postoperatively. These changes proved to be consistent in all subgroups during the whole follow-up period. Statistical differences appeared to be minimal and not significant **(**Table 4, Fig. 3**)**. Patients with therapy failure during the follow-up period were excluded from the calculations.

### Possible confounders

As the results concerning the primary endpoint indicated an age-related hip survival after treatment by means of core decompression, the distribution of possible confounders is provided for all age-dependent subgroups. The recorded parameters “lesion size”, “cam-type deformity” (on the basis of the alpha angle), “risk factors” and “treatment method” showed a homogenous distribution among the subgroups. This was confirmed in the corresponding statistical evaluation using the chi-square test with each *p* being far from significance **(**Table 5).

**Table 5:** Distribution of possible confounders among age-related subgroups in total numbers and rounded percentages

Possible confounder		Age-related ubgroups (count and percentage)			
		< 30 years	≥ 30/< 40 years	≥ 40/< 50 years	≥ 50 years
**Lesion Size**	< 15%	1 (11.1%)	2 (12.5%)	2 (7.4%)	2 (5.9%)
	≥ 15/≤ 30%	4 (44.4%)	8 (50%)	15 (55.6%)	22 (64.7%)
	> 30%	4 (44.4%)	6 (37.5%)	10 (37.0%)	10 (29.4%)
	Total	9 (100%)	16 (100%)	27 (100%)	34 (100%)
		***p*= 0.92**			
**Alpha angle**	< 50°	2 (22.2%)	3 (18.8%)	7 (25.9%)	8 (23.5%)
	≥ 50°/< 60°	3 (33.3%)	5 (31.3%)	7 (25.9%)	10 (29.4%)
	≥ 60°	4 (44.4%)	8 (50.0%)	13 (48.1%)	16 (47.1%)
	Total	9 (100%)	16 (100%)	27 (100%)	34 (100%)
		**p = 0.99**			
**Risk factor**	No	4 (44.4%)	8 (50.0%)	11 (40.7%)	19 (55.9%)
	Yes	5 (55.6%)	8 (50.0%)	16 (59.3%)	15 (44.1%)
	Total	9 (100%)	18 (100%)	27 (100%)	34 (100%)
		**p = 0.69**			
**Treatment type**	ACD	5 (55.6%)	7 (43.75%)	15 (55.6%)	19 (55.9%)
	mACD	4 (44.4%)	9 (56.25%)	12 (44.4%)	15 (44.1%)
	Total	9 (100%)	16 (100%)	27 (100%)	34 (100%)
		***p*= 0.86**			

## Discussion

Previous studies have shown the effect of certain parameters on the long-term outcome of ONFH therapy. These known parameters include lesion stage and lesion size as well as the presence of risk factors and concomitant hip pathologies [3, 21, 22].

As regards the parameter “age”, results show distinct differences for postoperative hip survival. Whereas the age-based subgroups “< 30 years” and “≥30 years/<40 years” presented a combined overall hip survival of 84%, older patients aged “≥40 years/<50 years” and “>50 years” had a significantly lower rate of only 54% of the combined hip survival over the study period. There is therefore a strong indication that at around the age of forty, there is a turning point in the long-term results of ONFH therapy.

It is known that bone healing in general and regeneration after ONFH strongly depends on the capacity of the individual’s body to repair and remodel the affected bone [30]. Although the underlying biochemical processes are not yet fully understood, a two-phase model consisting of a disintegrating osteoclast-driven phase and a bone-rebuilding osteoblast-driven phase is assumed [31]. Both phases are controlled by numerous growth and signal factors which balance the course of bone resorption and bone formation (e.g., bone morphogenetic proteins (BMP), tumour necrosis factor (TNF) and osteoprotegerin (OPG)) [32–34]. Basically, these reparative mechanisms are intended to work throughout life. Nevertheless, with increasing age, the human organism’s ability to make sufficient use of them seems to diminish. Recent studies have shown that fracture healing in elderly patients appears to take place rather slowly or with insufficient stability due to reduced mineral density [35–38]. It has also been shown that in vitro differentiation of bone marrow stroma cells to osteoblasts is less successful in cultures from older patients in comparison to those from younger patients [39].. Insufficiency of tissue regeneration in ONFH becomes even more apparent with regard to fibrosis. In this context, Sadile et al. evaluated the extension of histological fibrosis in a series of specimens from ONFH-biopsies. By proving a negative correlation, they were able to show that the extension of reactive fibrosis is a predictor of outcome of core decompression [40].

Regarding angioneogenesis, an important aspect in the healing process of ONFH, a decrease in regenerative potential can also be seen. As pluripotent bone marrow stroma cells decrease in number and potential for differentiation, the organism’s capacity to revascularize necrotic zones is highly limited. This condition is further aggravated by the aging of existing vessels, known as vascular aging, which is characterized by a progressive endothelial cell and smooth muscle cell dysfunction [41, 42].

Overall, it is known that the aging organism presents an increasing loss of functional tissue cells leading to an ongoing reduction in the ability of the tissues to maintain and replace themselves [43]. Therefore, it is not surprising that the success of ONHF therapy decreases with increasing age, and this is reflected in the rate of postoperative hip survival. The age of forty seems to mark a limit, with patients aged under forty having a significantly better chance of long-term hip survival after ACD, whereas about half of the patients older than 40 have to face therapy failure in the postoperative course. However, in this context, it must be noted that the short-term postoperative course seems to be similar in all patients, irrespective of age. As shown in Fig. 3, the Kaplan-Meier curves appear to be identical for the age groups < 40 and ≥ 40 years of age until the sixth month of the follow-up period **(**Fig. 2). Therefore, age-related factors affecting the overall therapy outcome seem to gain in importance only in the further postoperative course.

Concerning the parameter “sex”, it is known that the occurrence of ONFH is distributed unevenly between the sexes, affecting predominantly men [5, 6]. However, apart from its uneven distribution which might be due to still unknown sex-based factors according to Shimizu et al., sex as a parameter influencing the long-term outcome of ONFH-therapy had not previously been evaluated [23]. The present study indicates that patients’ sex seems to have no influence on therapy as the results of the calculated Kaplan-Meier estimator could not prove any significant sex-dependent differences in hip survival (*p* = 0.48). Therefore, it can be assumed that, based on current knowledge, the parameter “sex” is not relevant for the prediction of long-term ONFH therapy outcome.

However, as calculations revealed some differences in mean hip survival (male 51.3 months vs. female 61.4 months), the uneven distribution of the number of male and female study participants has to be taken into account. With only 20 female study patients, out of a total study collective of 86, statistical calculations might be biased and further examination with a greater number of female participants is required to confirm the study results. In this context, this study’s finding which indicates an earlier occurrence of treatment failure in the female subpopulation has to be verified as well.

As far as functional parameters and changes in pain levels are concerned, the study results appeared very uniform. A distinct amelioration of function of the affected hip, proved through a significant increase in mHHS from pre- to postoperative, was seen. A significant decrease in NRS demonstrated a clear improvement of postoperative pain levels. These findings were seen in the patients whose therapy was still successful after the follow-up period and were nearly identical, irrespective of the patient’s sex or age. Furthermore, this study’s results show consistent subgroup changes in mHHS and NRS at the different follow-up times. It follows that, in case of non-appearance of treatment failure, the process of rehabilitation is not affected by the parameters sex and age.

The results of the given study are limited by the fact that patient numbers of the investigated age groups were not evenly distributed. Therefore, an effect on the statistical analysis cannot be excluded. By proving a homogenous distribution of the possible confounders “lesion size”, “alpha angle”, “risk factors” and “surgery type” within the subgroups, an unwanted effect on the overall results was attempted to eliminate **(**Table 3**)**. However, as there are further possible confounders which could not be taken into account in the analysis (e.g. body mass index), a bias cannot be completely excluded [44]. Furthermore, it must be taken into account that the results presented in this study, although focused on the parameters “age” and “sex”, are based on the therapeutic intervention of core decompression. Although core decompression and its modifications are still widely used, its overall status in the treatment of ONFH has been repeatedly criticized. In their meta-analysis from 2016, Sadile et.al. were able to prove that especially classic core decompression does not improve clinical outcomes compared with other joint-preserving therapies. There even is some indication for less beneficial overall results [45]. On the whole, determining the role of core decompression remains difficult since lots of modifications have been described so far, and there is a lack of comparing studies. Hence, comparative analyses of the different surgical and non-surgical techniques regarding age- and sex-related outcome need to be undertaken in the future to further substantiate this study’s findings.

## Conclusion

The current study shows a distinct age-dependent rate of hip survival of ONFH patients treated by advanced core decompression. Therapy failure is significantly more likely to be seen in patients over 40 years of age. Patients’ sex does not seem to affect treatment outcome. For the patients who did not experience therapy failure during follow-up, postoperative clinical scores were independent of the parameters age and sex, and the rehabilitation process was not affected. Since the patient’s sex and age are unalterable parameters, the study cannot recommend any specific measures for the treatment of ONFH. However, it does show that therapy results are strongly age-dependent and thus it enables valuable predictions regarding the chances of long-term hip survival.

## Data Availability

The data that supports the findings of this study are available from the corresponding author upon reasonable request.

## Abbreviations

ONFH: Osteonecrosis of the femoral head
mHHS: Modified Harris Hip Score
NRS: Numeric rating scale
ACD: Advanced core decompression
mACD: Modified advanced core decompression
